# Fabricating Nanoporous Silica Structure on D-Fibres through Room Temperature Self-Assembly

**DOI:** 10.3390/ma7032356

**Published:** 2014-03-19

**Authors:** John Canning, Lucas Moura, Lachlan Lindoy, Kevin Cook, Maxwell J. Crossley, Yanhua Luo, Gang-Ding Peng, Lars Glavind, George Huyang, Masood Naqshbandi, Martin Kristensen, Cicero Martelli, Graham Town

**Affiliations:** 1 *i*nterdisciplinary Photonics Laboratories (*i*PL), School of Chemistry, the University of Sydney, Sydney, NSW 2006, Australia; E-Mails: lbarrosmoura@gmail.com (L.M.); lplindoy@gmail.com (L.L.); kevin.cook@sydney.edu.au (K.C.); shhu2628@uni.sydney.edu.au (G.H.); mnaq6951@uni.sydney.edu.au (M.N.); 2 School of Chemistry, the University of Sydney, Sydney, NSW 2006, Australia; E-Mail: maxwell.crossley@sydney.edu.au; 3 Graduate School of Electrical Engineering and Applied Computer Science, Federal University of Technology-Paraná, Curitiba PR 80230-901, Brazil; E-Mail: cmartelli@utfpr.edu.br; 4 Photonics & Optical Communications, School of Electrical Engineering and Telecommunications, the University of New South Wales, Sydney, NSW 2052, Australia; E-Mails: yanhua.luo1@unsw.edu.au (Y.L.); g.peng@unsw.edu.au (G.-D.P.); 5 Department of Engineering, Finlandsgade 22, Aarhus University, Aarhus N 8200, Denmark; E-Mails: lagla@vestas.com (L.G.); mk@eng.au.dk (M.K.); 6 Department of Engineering, Macquarie University, Sydney, NSW 2109, Australia; E-Mail: mnaq6951@uni.sydney.edu.au

**Keywords:** lab-in-a-fibre, lab-on-fibre, nanoparticles, biochemical, self-assembly, microfibers, filters, nanoreactions, sensors

## Abstract

The room temperature deposition of self-assembling silica nanoparticles onto D-shaped optical fibres (“D-fibre”), drawn from milled preforms fabricated by modified chemical vapour deposition (MCVD), is studied. Vertical dip-and-withdraw produces tapered layers, with one end thicker (surface coverage >0.85) than the other, whilst horizontal dip-and-withdraw produces much more uniform layers over the core region. The propagation of induced fracturing over the core region during drying is overcome using a simple protrusion of the inner cladding. Thick coatings are discernible through thin film interference colouring, but thinner coatings require scanning electron microscopy (SEM) imaging. Here, we show that fluorescence imaging, using Rhodamine B, in this example, can provide some qualitative and speedy assessment of coverage.

## 1. Introduction

It has recently been demonstrated how the self-assembly of nanoparticles can be used to fabricate directly novel optical microfibres at room temperature [1–3]. Controlled, room-temperature, evaporative self-assembly has overcome the thermodynamic barrier preventing the integration of many materials into silica. No better demonstration highlighted this than the integration of single photon emitting nanodiamonds impregnated with a nitrogen vacancy (NV) centre within silica [1], a process that could not be done otherwise given the high temperatures ordinarily required for processing silica, well above the annealing temperature of the NV centres. The properties of such fibres are in and of themselves quite interesting; the most recent work suggests, through a combination of direct local surface measurements using atomic force microscopy (AFM) and scanning electronic microscopy (SEM), and more indirect volume measurements using gas adsorption, extraordinarily uniform pore distribution, where a structure with hcp/fcc packing can have a classical crystal topography if the nanoparticles are sufficiently uniform in size [3]. Such pore distributions are potentially ideal for the needs of precision optical chromatography, molecular freezer, filter and sieve applications, as well as for advanced material design, such as future metamaterials [4]. Microfibre research is extremely attractive for these and many other applications, including enhanced sensitivity to surface and volume and chemical and physical interactions. However, their fragility beyond filtration, freezer and sieve applications has presented technical challenges for photonic interrogation and photonic device applications. In this work, we investigate room temperature self-assembly of layered structures directly onto D-shaped optical fibres. These fibres are the bedrock of optical sensing, the earliest designed exposed core fibres for enhancing sensing, whilst retaining robustness not much less than standard optical fibres, making the platform an ongoing favourite for field sensor work [5–7]. Many variants have since been developed, but the flat side of a D-shaped optical fibre (“D-fibre”) offers a unique platform onto which films and other devices can be deposited or assembled, even as an alternative substrate for the lithographic deposition of structures and layers, and has been used to deposit, for example, gold-doped porous gel silica layers [8]. They form one of the earliest forays into lab-in-a-fibre technologies [9,10], exploiting the inherent low cost per centimetre of a silica optical fibre.

Over the last few decades, substantive advances in layer-by-layer (LbL) deposition—polymeric, colloidal, nano- and micro-scale—has been demonstrated. A number of reports have shown techniques and apparatuses for coating different types of substrates with various materials in the form of thin layers [8,11–14]. Here, we report on the potential of deposition driven solely by room temperature convective self-assembly and intermolecular forces, the same processes used to fabricate the self-assembled optical microfibres. By using such a cold process, a much larger spectrum of material integration, particularly organic, is accessible. Ideally, however, rather than utilizing crack formation, which enables microfibre production (though this can also be done if appropriate), to produce smooth films, cracking needs to be removed altogether; this is a key challenge for many film deposition methods upon drying and is not unique to self-assembly. Further, the previous work on characterising self-assembled wires had run into a well-recognised problem in nanotechnology: the almost near absence of any rapid, convenient and routine diagnostic, even if only for the qualitative assessment of film presence. We also investigate here if fluorescence microscopy can provide some qualitative, rapid and useful assessment of film formation. In contrast to past work involving particle sizes sufficiently large for easy optical characterisation [15–19], the nanoscale is proving to be a real challenge with existing instrumentation operating far from real time.

## 2. Results and Discussion

### 2.1. D-Fibres

**Figure 1.**
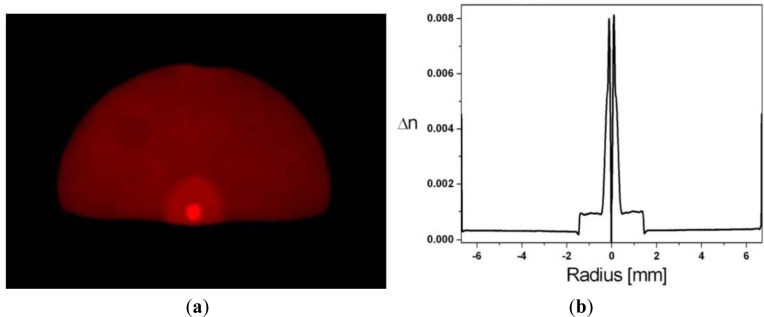
(**a**) Optical image of illuminated D-fibre cross-section; (**b**) corresponding refractive index profile of the preform.

The fibre used in this work is shown in Figure 1a and its refractive index profile in Figure 1b. A slight bulge is observed, due to material rounding, because the thermal expansion of the inner cladding differs from that of the outer cladding and was not considered during the draw, a factor that proves highly beneficial for this work. The properties of the particular fibre used in these experiments are summarised in Table 1. The fibre used had a measured core-flat distance of 8 μm to ensure that the study focuses on the surface effects of self-assembly on the inner cladding primarily.

### 2.2. Film Fabrication

**Figure 2.**
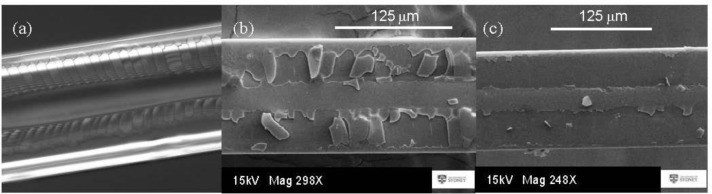
Horizontal “dip-and-withdraw” deposition of nanoparticle layers (stage *v* = 0.1 mm/s, *t*_stat_ = 5 min; [SiO_2_]_nano_ = 5 wt%). (**a**) Optical microscope image showing film formation with strong periodic cracking along the fibre; (**b**,**c**) SEM images at two points showing plates fracturing away in the cladding region where this cracking has occurred. The gun voltage, EHT, is typically 15kV. It is possible that the electron beam helped the plates to lift off the cladding, suggesting that the material over the core is more firmly bound.

Fabrication details for both horizontal and vertical dip-and-withdraw methods used to fabricate films can be found in the Experimental Section. Different velocities and nanoparticle concentrations provided mixed results, but overall, there is an observation of different degrees of self-assembly over the outer cladding and inner cladding/core regions. Of the two methods used, horizontal dip-and-withdraw leads to uniform layers over the core region, as shown in Figure 2a,b. However, these layers are much harder to control and are thicker; they can be observed easily enough in an optical microscope through typical film coloration, indicating that multiple layers are present. Further, the thicker layers built up in one step lead to strong cracking from the D-flat sides towards the centre, essentially arising when the solution begins contracting inwards through an analogous “coffee stain effect” [1–3,19] extended over the length of the fibre, where convective flow is impeded and stresses build up from packing. They usually (but not always) stop at the border of the inner cladding and are often highly periodic (Figure 2a; experimental conditions: stage velocity up/down *v* = 0.1 mm/s, sitting time *t* = 5 min; [SiO_2_]_nano_ = 5 wt%).

Beyond this, the film is observed under low-resolution scanning electron microscopy to be quite uniform over the core. This halting of crack propagation is indicative of significantly different surface interactions at the core and can, in part, be explained by the slight geometric protrusion of the inner cladding and core outwards from the D-flat, affecting convective flow and stress gradients beyond this point. It is an indication that controlled evaporation at slower rates may reduce or even avoid crack formation altogether outside the inner cladding, though difficult to implement. Some success in crack mitigation is obtained by adjusting conditions, although the coloration observed is a direct measure of film stresses, and the presence of variation indicates non-uniform thicknesses. Alternatively, the induced stresses are sufficiently uniform along the fibre, that the fracturing that takes place is periodic, and this could be of use in novel devices, since it is in these regions where post-doping with functional species will likely accumulate most. In the SEM images, it appears that the removal of the layers is occurring only in the outer cladding region, whereas the core region seems intact. This might suggest that surface attachment is stronger at the core and inner cladding regions (where surface defect sites, for example, are going to be in much higher concentrations and wetting properties will differ), or more likely, stresses and packing are higher on the latter planes and, therefore, fracturing far more likely. The high current and exposure time of the 15-kV electron beam may also be sufficient to induce these plates to lift off.

**Figure 3.**
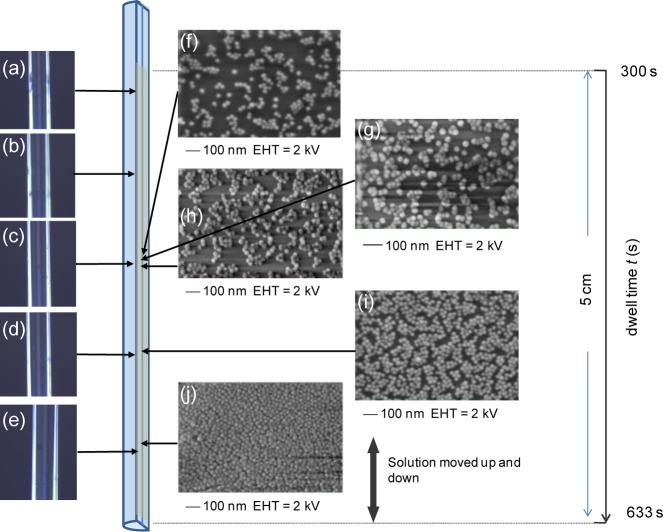
Vertical “dip-and-withdraw” deposition of nanoparticle layers: (**a**) stage *v* = 0.3 mm/s, *t*_0_ = 300 s (5 min); (SiO_2_)_nano_ = 7.5 wt%. Optical microscope images on the left (**a**–**e**) showing no clear sign of thick films (thin film interference is seen in Figure 4b with no cracking at this velocity). SEM analysis along the fibre (EHT = gun voltage), on the right (**f**–**j**), shows partial coating down the fibre with complete coverage (>85%) at the end of the fibre, where the overall longest immersion period (dwell time) was spent; (**g**–**h**) are taken, within experimental error, over a region ~1 mm-long.

In contrast to the horizontal approach, vertical dip-and-withdraw at constant velocity leads to tapered layers, because the end of the fibre spends more time immersed than further along the fibre. This is illustrated in Figure 3. In these experiments, results were obtained for a stage velocity of 0.3 mm/s, resulting in total dwell times over a 5-cm length varying from *t*_0_ = 300 s to *t* = 633 s, more than double the rest dwell time, *t*_0_. The higher velocity was found to reduce crack generation in the vertical dip-and-withdraw method. In contrast to the previous results, optical images showed little strong film interference, indicating the layers, if present, are much thinner.

The total surface coverage as a percentage, determined from the SEM images on the right (obtained at lower electron beam energies of 2 kV to reduce the likelihood of any beam ablation), clearly varies. On a local millimetre, scale the variation is ~(0.31–0.45) in Figure 3g–h, reaching ~0.65 in Figure 3i and >0.85 towards the end of the fibre in Figure 3j (where *t* ~ 550 s), as determined by a software 2D fitting routine. Visual inspection would say higher (close to one), so these higher values need to be considered with caution. Nonetheless, the trend towards increasing surface coverage with longer dwell time is clear. Figure 3 shows the importance of this time on the uniformity of coverage, as well as permitting a precise total dwell time for the deposition to be extracted for a given concentration (here, [SiO_2_]_nano_ = 7.5 wt%). The measurements are taken about 1 cm apart, except in Figure 3g–h, taken over ~1 mm to determine local variations. Generally, there is some uncertainty with the exact positions of the SEM in this data relative to the optical images.

By adjusting the velocity of the stage through acceleration, the variation in dwell time can be reduced and the tapering varied. The vertical approach also allows much greater finesse and crack mitigation by building up layers through multiple passes. This is analogous to the approach used in fabricating sol-gel glass waveguides by building thick layers from thin layers, where the threshold for crack propagation is not exceeded [20].

### 2.3. Fluorescence Microscopy of Films on D-Fibre

Three dye-treated samples were prepared as described in the Experimental Section. Just coating the fibre with 9-(2-carboxyphenyl)-6-diethylamino-3-xanthenylidene]-diethylammonium chloride (Rhodamine B), saw little attachment and little colouring of the optical fibre. On the other hand, coating a sample with a self-assembled film showed clear colouring from percolation into the film. Integrating the dye during film formation introduced much higher concentrations of dye, which affected film formation. The discrimination between fibres with and without films is clear, offering a potentially simple optical means of assessing film quality by monitoring fluorescence.

From other work [4], we know that percolation of Rhodamine B dye into self-assembled wires is rapid, demonstrating signs of super, or ballistic, diffusion in contrast to many other organic species; so, similar percolation is expected in Sample 2. It can be concluded that the total silica surface area covered by this means is much larger within the layer compared to having no layer at all. If similar hcp or fcc packing to the self-assembled wires is assumed (which is not unreasonable, given that the same intermolecular forces are present), then the internal volume of the layer is ~26% of the total layer (variations arising from particle size distribution not withstanding). From adsorption measurements of the wires [3], the surface area per gram of material can be assumed to be ~100 m^2^/g. This corresponds to a very large increase in effective surface area, readily accounting for the considerable difference in emission between samples.

**Figure 4.**
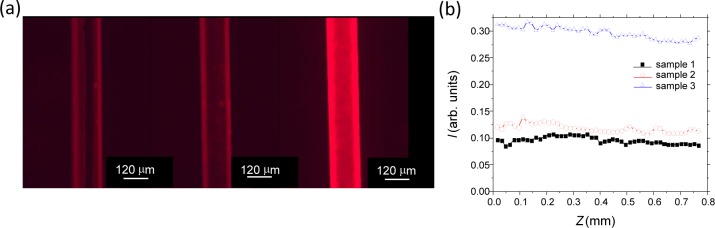
(**a**) Fluorescence image of Samples 1–3 (left to right) and (**b**) line intensities over the fibre core region (Sample 1, ■; Sample 2, **○**; Sample 3, **Δ**).

Of the three samples described in the experimental, Sample 3 clearly is the brightest under ambient lighting conditions to the naked eye. Figure 4a shows the red fluorescence microscopy image of each sample when directly excited by green light. As expected from the optical images, Sample 1 shows the least fluorescence, whereas both Samples 2 and 3 show more fluorescence, the latter being noticeably brighter.

These values can be readily quantified. Figure 4b shows the average intensities of the photographs taken along a small region over the core of each sample. The data is normalized to a saturation value of one. These intensities correlate with the variable absorption, or colouring, seen in the photographs and indicate the amount of trapped Rhodamine B on the fibres. Sample 2 shows a little more than Sample 1 (~20% increase), whereas Sample 3, where silica is mixed with dye before film formation, shows more than three times this. This suggests the latter has produced thicker layers, possibly thick enough to also trap light and increase the apparent intensity observed. It can also be noticed that the red is not constant, suggesting significant surface topology variation.

**Figure 5.**
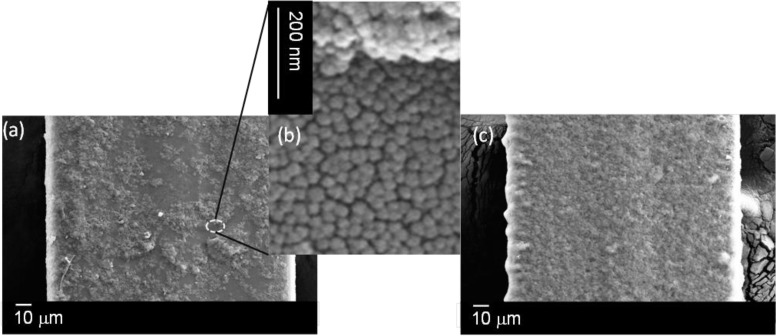
SEM images of (**a**) Sample 2 and inset (**b**) of Sample 2 showing the assembled layer below aggregated regions; and (**c**) Sample 3.

In order to better understand the origin of these differences and explore any surface topology, the films on Samples 2 and 3 were examined under SEM. Figure 5a–c shows the overall SEM images for both samples. Close examination reveals that there are three distinct regions visible for Sample 2: a uniform thin film layer with very little surface variation suggesting good film formation initially (close-up in Figure 5b), probably only a few layers thick at best (<100 nm), followed by a layer where there is less tidy, or random, packing, with particles appearing spread out over the first layer and, finally, the aggregating and clumping of particles with very high surface topology (Figure 5a). Thus we identify three types of deposition processes. The first appears to be uniform and, all over the fibre, flat; but, because they are on the nanoscale (<100 nm), they cannot be detected optically (no thin film interference). On the other hand, they give rise to the 20% or so increase in the fluorescence signal observed (where the data in Figure 4b was taken from the core region where the fluorescence was most uniform). The second regime, where additional particles appear intermittent and less regular on the surface, is not so uniform (Figure 5a). Then, there are significant clumps or aggregates of particles in no specific order, although closely packed within, and these are clearly much thicker: the amount of this material increases with the number of passes, suggesting that two or less passes are required to obtain a reasonably well-packed mono-layer or a few layers. In the fluorescent images, the regimes where surface topology varies give rise to the brighter fluorescence spots that appear.

For Sample 3, the SEM data show that the surface is almost fully covered by a less regular coating with variable surface topology (Figure 5c). This indicates significantly improved overall coverage, but mainly in the third regime of deposition identified for Sample 2, almost as if the aggregations are filling the gaps. This region is easily observed in visible silica layers with no dye as strong thin film interference colours. When examined closely, the layer appears to be undulating like dimples in regions, and there are additional distributed pores. The general impression is much more of an amorphous and porous, glassy structure than crystalline; the added porosity is larger than that expected from a densely packed crystal film, and so, this will also help to explain the significantly stronger accumulation of Rhodamine B into these films. It also accounts for the patchy red appearance of the fluorescence image of Figure 4a on the far right. The formation of these thicker layers may be encouraged by the reduced role of dispersion forces if the dye coverage is sufficient to prevent the optimal proximity for attractive forces to dictate self-assembly. In any case, Rhodamine B mixed with the silica nanoparticles prior to deposition appears to encourage much thicker formation and aggregation of layers. Given that it has a carboxylic end group, it is likely to be weakly attaching to both the silica fibre surface and to the nanoparticles. Some evidence of this is found by the fact that it is relatively easy to remove these thicker layers.

**Figure 6.**
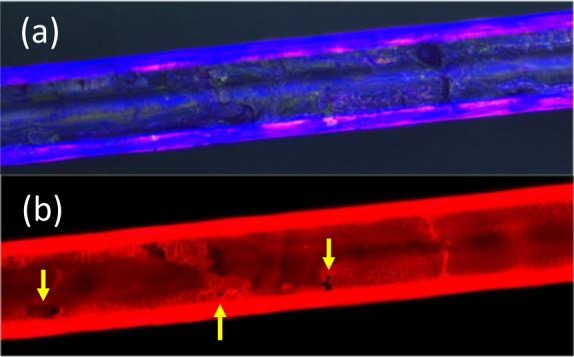
Images of one sample of D-shaped fibre cracking due to handling and bending: (**a**) optical micrograph showing intense interference colours and (**b**) fluorescence image after adding Rhodamine B. Cracks and defects are readily visible. In small regions where the layer has come off, these are dark and marked with yellow arrows.

Overall, fluorescence from Rhodamine B added to the silica layers helps to improve an optical assessment of the layer formation. SEM analysis confirms the presence of nanoparticles everywhere. As suggested by the first optical images, in samples coated with silica dye, it can be added to highlight those areas that are indeed coated and those that are not. Fluorescence contrast between uncoated regions and coated regions is clear; an example where a coating is not uniform despite large colouring from thin film interference is shown in Figure 6. The Rhodamine B also highlights bend-induced cracking arising from the handling of the sample. Notably, it does not necessarily correlate with areas on the optical image that suggest a hole is present. Part of this added illumination stems from light trapped by thicker layers, as well as the optical fibre itself (which is why the edges are saturated).

## 3. Experimental Section

### 3.1. D-Fibres

These were fabricated at the national Fibre Facility at The University of New South Wales. Typically, 15 passes on a modified chemical vapour deposition (MCVD) lathe were used to build up the inner silica cladding inside a standard Heraeus silica tube followed by a core layer of germanate and silicate. The preform produced was consolidated ~1900 °C and then milled into a D-shape, where one side is flat. A refractive index profile was measured and scaled down according to optical fibre dimensions. This was drawn in stages on a customised draw tower to balance outer diameters, single mode operation and core-to-flat distances. All optical images were taken using a standard optical microscope with a 25× objective.

### 3.2. Silica Nanoparticles

**Figure 7.**
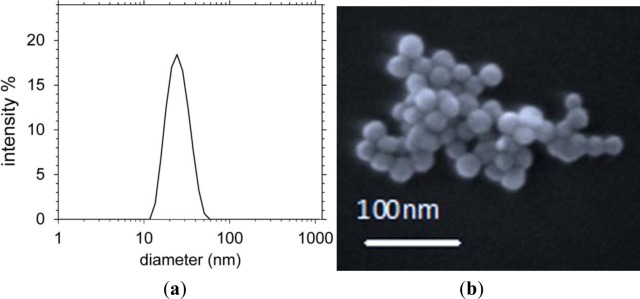
(**a**) Dynamic light scattering (DLS) measurements of silica particle size distribution in H_2_O and (**b**) an SEM image of some of the nanoparticles on a slide.

Commercially available colloidal silica nanoparticles in water (40% w/w, H_2_O, pH = 9) were diluted to 2.5 wt% for this work. The size distribution was determined using dynamic light scattering (DLS) and scanning electronic microscopy (SEM) to be within 20–30 nm in diameter (shown in Figure 7). The size of these particles is obviously much smaller than those required to self-assemble opal-like crystals with fcc packing for near-infrared and visible applications.

### 3.3. Deposition onto D-Fibre

Various methods for depositing the nanoparticles onto the D-shaped fibre were explored.

**Figure 8.**
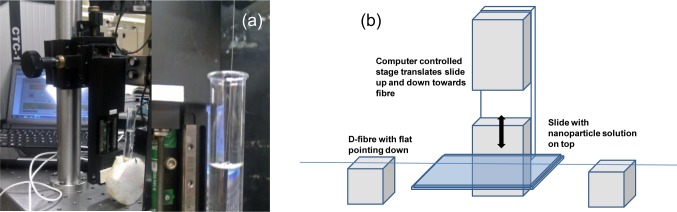
Dip-and-withdraw deposition of nanoparticles: (**a**) vertical; (**b**) horizontal.

For the initial film work, both horizontal and vertical dip-and-withdraw were explored. These setups are shown in Figure 8.

**Figure 9.**
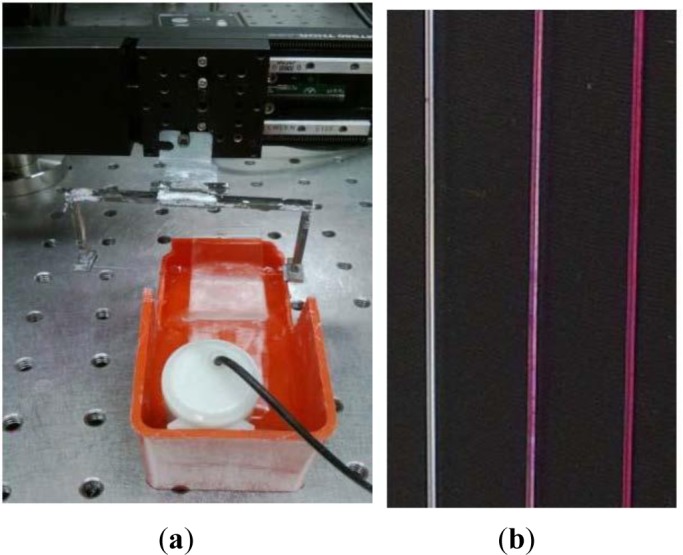
(**a**) Setup in use, with the miniature pump moving the solution onto the slide on which the fibre is scanned horizontally by the automated translation stage; (**b**) the three treated Samples 1–3 (left to right).

For the fluorescence work reported here, the best results were obtained using an automated horizontal scan, where the fibre sample is moved horizontally back and forth using a motorized stage through the desired solution on a glass slide. A miniature pump is used to ensure that the solution remains in supply on the stage during the process. Figure 9a shows the simple setup used. The samples were then analysed using both optical and fluorescence microscopy under identical conditions, where the dye is excited with green light and the red emission imaged and compared across samples. For fluorescence, all samples were analysed on a black surface, and the images presented in the results were taken with a ×10 objective, 180 ms exposure time and zero CCD (charged coupled device) gain.

### 3.4. Fluorescence Studies

Water soluble Rhodamine B (C_28_H_31_O_3_N_2_Cl) was used: the dye concentration was 2.19 g/L. Three fibre samples were prepared:

Sample 1: D-fibre with dye solution only scanned 10 times at 0.3 mm/s (control).

Sample 2: D-fibre with self-assembled layers of nanoparticles with 0.5 wt% NaCl salt and then dye solution scanned 10 times at 0.3 mm/s.

Sample 3: D-fibre with self-assembled layers of nanoparticles with NaCl salt and dye solution scanned 10 times at 0.3 mm/s.

Images of the three scans are shown in Figure 9b. When present, the Rhodamine B is visible as a pink colouring. From this image, it can be seen that very little dye is visible in Sample 1, so simply coating the D-fibre shows very little attachment. By contrast, when silica nanoparticle coatings are present, the dye is clearly visible.

Some of the experimental conditions and experimental parameters are summarised in Table 2.

## 4. Conclusions

The cold deposition of silica nanoparticle layers on D-shaped optical fibres by convective flow-based self-assembly was investigated. A clear interdependence on concentration, velocity and dwell time was found. There appears to be a threshold cracking thickness or concentration level, which can be mitigated using higher velocities to form thinner layers. In contrast to horizontal immersion, using constant velocity, strongly tapered films are produced of varying thickness with vertical immersion. In this respect, the vertical dip-and-withdraw method allows greater control and refinement in identifying optimal conditions, because of the chirped exposure time along the fibre, overcoming the need for multiple scans at different velocities to achieve similar results.

Generally, stress fractures are observed to occur from the outside in, and these stop at the inner cladding boundary; this occurs because the geometric protrusion of the inner cladding acts to impede laminar flow beyond that point and, in effect, impedes crack propagation over the core. It offers a relatively simple means of reducing crack propagation and reducing the time needed to build up the film using layer-by-layer approaches.

The horizontal approach allows for more uniform layers. By combining both vertical and horizontal flow, complex film designs are possible as a consequence of the greater flexibility in varying and controlling layer thickness. Clearly, the methods described here apply to any surface coverage and are not limited to a singular particle component or to D-shaped fibres. Future work, amongst many things, will explore optical planar devices, including photonic crystal waveguides for biosensing and novel quantum sources [21–23].

Towards this end, scanning was introduced into the horizontal dip-and-withdraw process to allow for greater film control. The integration of a fluorescence dye during nanoparticle self-assembly is observed to enhance film formation, and different deposition regimes from a few uniform layers to thicker aggregated layers are observed. The dye tends to reduce cracking, although this appears to come at the expense of film robustness and order, given the larger quantity of dye that is incorporated compared to post-addition; more work is needed to assess both the potential and the limitations of this aspect. On the other hand, the dye can enhance the appearance of cracks to provide rapid diagnostics on the quality of the film when added after film formation. SEM, whilst useful, is, by contrast, a time-consuming process that is not yet routine, because it has such a localised angle of view. Tools are needed for quick routine inspection over a sufficiently large field of view, and fluorescence microscopy shows promise in contributing towards addressing this goal.

## Figures and Tables

**Table 1.** Measured parameters of the D-fibre.

ϕ_fibre_ (μm)	ϕ_core_ (μm)	d (core to flat) (μm)	n_core_	n_clad_	MFR (mode field radius, μm)
125	5.7	8	1.466	1.461	6.25

D-Fibre fabricated by modified chemical vapour deposition (MCVD).

**Table 2.** Summary of the conditions of the experiments and solutions used.

D-fibres	Horizontal Scan							
	1st round of scans				2nd round of scans			
	Solution	Scans #	Dwell time (s)	Velocity (mm/s)	Solution	Scans #	Dwell time (s)	Velocity (Mm/s)
**Sample 1**	2.19 g/L Rhodamine B	10	0	0.3	N/A	N/A	N/A	N/A

**Sample 2** *	2.5 wt% silica + 0.5 wt% NaCl	10	0	0.3	N/A	N/A	N/A	N/A
	N/A	N/A	N/A	N/A	2.19 g/L Rhodamine B	10	0	0.3

**Sample 3**	2.5 wt% silica + Rhodamine B 2.19 g/L + 0.5 wt% NaCl	10	0	0.3	N/A	NA	N/A	N/A

* Sample 2 was scanned in Rhodamine B after being scanned in silica nanoparticles solution.
